# Supplementary material for: The impact of temporal resampling on the safety of clinical reinforcement learning: A simulated trial
Source: PLOS Digit Health. 2026 Nov 6;5(11):e0001801. doi: 10.1371/journal.pdig.0001801 (PMC13743037; doi:10.1371/journal.pdig.0001801)
Supplement: Supplementary file 1 [file pdig.0001801.s007.pdf]

S1 Table. Literature Review: Summary of relevant literature from 2016 to 2026 for offline reinforcement learning using electronic health record data, including choice of resampling interval where applicable.

Paper	Date	Authors	Title	Data	Model	Evaluation	Intervals
**1**	2016 Aug	Nemati et al. [71]	Optimal medication dosing from suboptimal clinical examples	MIMIC-II	Fitted Q-Iteration (model-free)	Retrospective evaluation only	1 hour
**2**	2017 Aug	Prasad et al. [12]	A reinforcement learning approach to weaning of mechanical ventilation in intensive care units	MIMIC-III	Fitted Q-Iteration (model-free)	Retrospective evaluation only	10 minutes
**3**	2017 Nov	Raghu et al. [47]	Continuous state-space models for optimal sepsis treatment: a deep reinforcement learning approach	MIMIC-III	Dueling Double Deep Q-Network (model-free)	Retrospective evaluation only	4 hours
**4**	2018 Jul	Lin et al. [72]	A deep deterministic policy gradient approach to medication dosing and surveillance in the ICU	MIMIC-III, Emory University	Deep Deterministic Policy Gradient (model-free)	Retrospective evaluation only	Unspecified (3 hours?)
**5**	2018 Jul	Wang et al. [73]	Supervised reinforcement learning with recurrent neural network for dynamic treatment recommendation	MIMIC-III	Supervised RL with Recurrent Neural Network (model-free)	Retrospective evaluation only	24 hours
**6**	2018 Nov	Komorowski et al. [7]	The artificial intelligence clinician learns optimal treatment strategies for sepsis in intensive care	MIMIC-III, eICU	Policy Iteration (model-based)	Retrospective evaluation only	4 hours
**7**	2018 Dec	Peng et al. [48]	Improving sepsis treatment strategies by combining deep and kernel-based reinforcement learning	MIMIC-III	Dueling Double Deep Q-Network (model-free)	Retrospective evaluation only	4 hours
**8**	2019 Feb	Liu et al. [74]	Learning the dynamic treatment regimes from medical registry data through deep Q-network	CIBMTR	Deep Q-Network (model-free)	Retrospective evaluation only	5 total bins
**9**	2019 Jul	Lopez et al. [75]	Deep reinforcement learning for optimal critical care pain management with morphine using dueling double-deep Q networks	MIMIC-III	Dueling Double Deep Q-Network (model-free)	Retrospective evaluation only	1 hour
**10**	2019 Apr	Yu et al. [76]	Inverse reinforcement learning for intelligent mechanical ventilation and sedative dosing in intensive care units	MIMIC-III	Inverse RL with Fitted Q-Iteration (model-free)	Retrospective evaluation only	10 minutes
**11**	2020 May	Futoma et al. [77]	Identifying distinct, effective treatments for acute hypotension with SODA-RL: Safely optimized diverse accurate reinforcement learning	MIMIC-III	SODA-RL (model-free)	Retrospective evaluation only	1 hour
**12**	2020 July	Tang et al. [49]	Clinician-in-the-loop decision making: reinforcement learning with near-optimal set-valued policies	MIMIC-III	Set-Valued Policy (model-free)	Retrospective evaluation only	4 hours
**13**	2020 Jul	Yu et al. [78]	Supervised-actor-critic reinforcement learning for intelligent mechanical ventilation and sedative dosing in intensive care units	MIMIC-III	Supervised Actor-Critic (model-free)	Retrospective evaluation only	10 minutes

Paper	Date	Authors	Title	Data	Model	Evaluation	Intervals
**14**	2020 Nov	Lu et al. [36]	Is deep reinforcement learning ready for practical applications in healthcare? A sensitivity analysis of duel-DDQN for hemodynamic management in sepsis patients	MIMIC-III	Dueling Double Deep Q-Network (model-free)	Retrospective evaluation only	1 hour
**15**	2020 Dec	Killian et al. [50]	An empirical study of representation learning for reinforcement learning in healthcare	MIMIC-III	Batch-Constrained Q-Learning (model-free)	Retrospective evaluation only	4 hours
**16**	2021 Feb	Peine et al. [79]	Development and validation of a reinforcement learning algorithm to dynamically optimize mechanical ventilation in critical care	MIMIC-III, eICU	Q-Learning (model-free)	Retrospective evaluation only	4 hours
**17**	2021 Feb	Roggeveen et al. [51]	Transatlantic transferability of a new reinforcement learning model for optimizing haemodynamic treatment for critically ill patients with sepsis	MIMIC-III, Amsterdam UMCdb	Dueling Double Deep Q-Network (model-free)	Retrospective evaluation only	4 hours
**18**	2021 Mar	Eghbali et al. [80]	Patient-specific sedation management via deep reinforcement learning	MIMIC-IV	Deep Deterministic Policy Gradient (model-free)	Retrospective evaluation only	1 hour
**19**	2021 Aug	Sun et al. [81]	Personalized vital signs control based on continuous action-space reinforcement learning with supervised experience	MIMIC-III	Deep Deterministic Policy Gradient (model-free)	Retrospective evaluation only	1 hour
**20**	2021 Nov	Liu et al. [52]	Combining model-based and model-free reinforcement learning policies for more efficient sepsis treatment	MIMIC-III	Policy Iteration + Dueling Deep Q-Network (model-based and model-free)	Retrospective evaluation only	4 hours
**21**	2021 Dec	Fatemi et al. [9]	Medical dead-ends and learning to identify high-risk states and treatments	MIMIC-III	Customised Double Deep Q-Networks (model-free)	Retrospective evaluation only	4 hours
**22**	2021 Dec	Satija et al. [53]	Multi-objective SPIBB: Seldonian offline policy improvement with safety constraints in finite MDPs	MIMIC-III	Safe Policy Improvement with Baseline Bootstrapping (model-based)	Retrospective evaluation only	4 hours
**23**	2022 Jan	Yala et al. [82]	Optimizing risk-based breast cancer screening policies with reinforcement learning	Massachusetts General Hospital	Envelope Q-Learning (model-free and model-based)	Retrospective evaluation only	6 months
**24**	2022 Feb	Guo et al. [83]	Learning dynamic treatment strategies for coronary heart diseases by artificial intelligence: real-world data-driven study	MIMIC-III	Deep Deterministic Policy Gradient (model-free)	Retrospective evaluation only	24 hours
**25**	2022 Feb	Li et al. [84]	Electronic health records based reinforcement learning for treatment optimizing	MIMIC-III	Deep Q-Network + Model-Based RL (model-free and model-based)	Retrospective evaluation only	3 hours

Paper	Date	Authors	Title	Data	Model	Evaluation	Intervals
**26**	2022 Feb	Nanayakkara et al. [85]	Unifying cardiovascular modelling with deep reinforcement learning for uncertainty aware control of sepsis treatment	MIMIC-III	Distributional RL (model-free)	Retrospective evaluation only	1 hour
**27**	2022 Apr	Prasad et al. [86]	Guiding efficient, effective, and patient-oriented electrolyte replacement in critical care	UPHS, MIMIC-IV	Fitted Q-Iteration (model-free)	Retrospective evaluation only	6 hours
**28**	2022 Apr	Su et al. [87]	Establishment and implementation of potential fluid therapy balance strategies for ICU sepsis patients based on reinforcement learning	PICMISD	Deep Q-Network (model-free)	Retrospective evaluation only	6 hours
**29**	2022 Aug	Huang et al. [88]	Reinforcement learning for sepsis treatment: a continuous action space solution	MIMIC-III	Deep Deterministic Policy Gradient (model-free)	Retrospective evaluation only	4 hours
**30**	2022 Sep	Shiranthika et al. [11]	Supervised optimal chemotherapy regimen based on offline reinforcement learning	Local data	Conservative Q-Learning (model-free)	Retrospective evaluation only	**Already regular**
**31**	2023 Feb	Wu et al. [37]	A value-based deep reinforcement learning model with human expertise in optimal treatment of sepsis	MIMIC-III	Weighted Dueling Double Deep Q-Network (model-free)	Retrospective evaluation only	1, 2, 4, 6, and 8 hours
**32**	2023 May	Gao et al. [16]	Offline learning of closed-loop deep brain stimulation controllers for Parkinson disease treatment	Local	Deep Deterministic Policy Gradient (model-free)	**Retrospective evaluation + clinical trial**	**Already regular**
**33**	2023 May	Liang et al. [54]	The treatment of sepsis: an episodic memory-assisted deep reinforcement learning approach	MIMIC-III	Dueling Double Deep Q-Network (model-free)	Retrospective evaluation only	4 hours
**34**	2023 June	Kondrup et al. [13]	Towards safe mechanical ventilation treatment using deep offline reinforcement learning	MIMIC-III	Conservative Q-Learning (model-free)	Retrospective evaluation only	4 hours
**35**	2023 Oct	Wang et al. [14]	Optimized glycemic control of type 2 diabetes with reinforcement learning: a proof-of-concept trial	Zhongshan Hospital, Qingpu Hospital	Customised Model-based RL (model-based)	**Retrospective evaluation + clinical trial**	7 daily bins
**36**	2023 Oct	Zhu et al. [18]	Offline deep reinforcement learning and off-policy evaluation for personalized basal insulin control in type 1 diabetes	UVA/Padova T1DM, OhioT1DM	TD3-BC (model-free)	Retrospective evaluation + simulated trial	**Already regular**
**37**	2024 Jan	den Hengst et al. [89]	Guideline-informed reinforcement learning for mechanical ventilation in critical care	MIMIC-III	Q-Learning, Imitation Learning (model-free)	Retrospective evaluation only	4 hours
**38**	2024 Jan	Lee et al. [90]	Reinforcement learning model for optimizing dexmedetomidine dosing to prevent delirium in critically ill patients	SNUH	Conservative Q-Learning (model-free)	Retrospective evaluation only	6 hours
**39**	2024 Feb	Petch et al. [33]	Optimizing warfarin dosing for patients with atrial fibrillation using machine learning	ARISTOTLE, ENGAGE AF-TIMI, ROCKET AF, RE-LY	Batch-Constrained Q-Learning (model-free)	Retrospective evaluation only	**Not used**

Paper	Date	Authors	Title	Data	Model	Evaluation	Intervals
**40**	2024 Mar	Zhang et al. [91]	Optimizing sepsis treatment strategies via a reinforcement learning model	MIMIC-III	Dueling Double Deep Q-Network (model-free)	Retrospective evaluation only	2 hours
**41**	2024 Apr	Job et al. [30]	Optimal treatment strategies for critical patients with deep reinforcement learning	MIMIC-III	Dueling Double Deep Q-Network (model-free)	Retrospective evaluation only	**Not used**
**42**	2024 Aug	Beolet et al. [19]	End-to-end offline reinforcement learning for glycemia control	UVA/Padova T1DM	BCQ, TD3-BC, CQL (model-free)	Simulated trial only	1 hour
**43**	2025 Feb	Ghasemi et al. [32]	Personalized decision making for coronary artery disease treatment using offline reinforcement learning	APPROACH	Q-Learning, Deep Q-Learning, Conservative Q-Learning (model-free)	Retrospective evaluation only	**Not used**
**44**	2025 Feb	Tu et al. [55]	Offline safe reinforcement learning for sepsis treatment: tackling variable-length episodes with sparse rewards	MIMIC-III	Conservative Q-Learning, Double Deep Q-Network (model-free)	Retrospective evaluation only	4 hours
**45**	2025 May	Desman et al. [92]	A distributional reinforcement learning model for optimal glucose control after cardiac surgery	MIMIC-IV, eICU	Distributional RL + Conservative Q-Learning (model-free)	Retrospective evaluation only	1 hour
**46**	2025 May	Kalimouttou et al. [93]	Optimal vasopressin initiation in septic shock: the OVISS reinforcement learning study	MIMIC-IV, eICU, UPMC, UCSF	Fitted Q-Iteration (model-free)	Retrospective evaluation only	1 hour
**47**	2025 Aug	Zhou et al. [31]	Optimizing long term disease prevention with reinforcement learning: a framework for precision lipid control	HKHA	Policy Iteration (model-based)	Retrospective evaluation only	**Not used**
**48**	2025 Sep	Fang et al. [56]	Offline inverse constrained reinforcement learning for safe-critical decision making in healthcare	MIMIC-III	Constraint Transformer (model-based)	Retrospective evaluation only	4 hours
**49**	2025 Oct	Lee et al. [94]	Optimizing Loop Diuretic Treatment for Mortality Reduction in Patients With Acute Dyspnea Using a Practical Offline Reinforcement Learning Pipeline for Health Care: Retrospective Single-Center Simulation Study	Michigan Medicine	Batch-Constrained Q-Learning, Pessimistic MDP (model-free and model-based)	Retrospective evaluation only	24 hours
**50**	2026 Feb	Fan et al. [17]	Reinforcement learning-based digital therapeutic intervention for postprostatectomy incontinence: development and pilot feasibility study	Local	Q-learning (model-free)	**Retrospective evaluation + clinical trial**	1 hour
