# Supplementary material for: The impact of temporal resampling on the safety of clinical reinforcement learning: A simulated trial
Source: PLOS Digit Health. 2026 Nov 6;5(11):e0001801. doi: 10.1371/journal.pdig.0001801 (PMC13743037; doi:10.1371/journal.pdig.0001801)
Supplement: Supplementary file 2 [file pdig.0001801.s008.pdf]

S2 Table. REFORMS checklist: As per Kapoor et al. [70].

Item	Description	Page	Notes
**Module 1: Study goals**			
1a	Population or distribution about which the scientific claim is made.	4	Offline reinforcement learning on any temporally resampled healthcare data.
1b	Motivation for choosing this population or distribution (1a.).	4	Explore risks to patients from decision models trained on resampled (rather than unprocessed) data.
1c	Motivation for the use of ML methods in the study.	3	Clinical reinforcement learning could improve healthcare decision models, but often mandates an offline training approach.
**Module 2: Computational reproducibility**			
2a	Dataset used for training and evaluating the model along with link or DOI to uniquely identify the dataset.	13	Datasets were generated uniquely by interacting with the LavaGap and UVA/Padova environments. Available at the GitHub repo below.
2b	Code used to train and evaluate the model and produce the results reported in the paper along with link or DOI to uniquely identify the version of the code used.	17	Code, datasets, instructions all publicly available at the associated GitHub repo.
2c	Description of the computing infrastructure used.	17	Python (v3.10), PyTorch (v2.9.0), Gymnasium (0.29.1), Numba-accelerated SimGlucose. (See Methods).
2d	README file which contains instructions for generating the results using the provided dataset and code.	17	Code, datasets, instructions all publicly available at the associated GitHub repo.
2e	Reproduction script to produce all results reported in the paper.	17	Code, datasets, instructions all publicly available at the associated GitHub repo.
**Module 3: Data quality**			
3a	Sources of data, separately for the training and evaluation datasets (if applicable), along with the time when the datasets are collected, the source and process of ground-truth annotations, and other data documentation.	13	Expert PPO policy used to generate 100k transitions (LavaGap) and 10M transitions (UVA/Padova), which are processed as described in the Methods.
3b	Distribution or set from which the dataset is sampled (i.e., the sampling frame).	13	As described in the methods.
3c	Justification for why the dataset is useful for the modeling task at hand.	4	Generating from a clinically validated simulator enables quick, safe prospective evaluation of the models.
3d	The definition of the outcome variable of the model along with descriptive statistics, if applicable.	16	The interquartile mean of episode returns with 95% confidence intervals.
3e	Number of samples in the dataset.	14	100,000 transitions (LavaGap); 10,000,000 transitions (UVA/Padova).
3f	Percentage of missing data, split by class for a categorical outcome variable.	14	0% missing data (generated from simulated environments).
3g	Justification for why the distribution or set from which the dataset is drawn (3b.) is representative of the one about which the scientific claim is being made (1a.).	6	Datasets are drawn from environments with irregular decision intervals. They can then be resampled prior to training.
**Module 4: Data preprocessing**			
4a	Identification of whether any samples are excluded with a rationale for why they are excluded.	12	UVA/Padova: 12 patients excluded from training — 6 for validation, 6 for testing.

Item	Description	Page	Notes
4b	How impossible or corrupt samples are dealt with.	12	N/A for LavaGap. For UVA/Padova, episodes terminated early if glucose < 10 mg/dL or > 600 mg/dL (with a $-10,000$ penalty).
4c	All transformations of the dataset from its raw form (3a.) to the form used in the model, for instance, treatment of missing data and normalization.	14	Temporal binning (2h/4h), interpolation (10 mins), and reward standardization to zero mean/unit variance.
**Module 5: Modeling**			
5a	Detailed descriptions of all models trained, including:	15	BC, IQL, and CQL algorithms trained using identical neural networks across three dataset variants (unprocessed, binned, interpolated).
	All features used in the model (including any feature selection).	12	LavaGap: partial grid pixels. UVA/Padova: blood glucose, insulin rate, time of day, and carbohydrate intake.
	Types of models implemented (e.g., Random Forests, Neural Networks).	15	LavaGap: CNN encoder with MLP heads. UVA/Padova: MLP + LSTM encoder with MLP heads.
	Loss function used.	15	Mean squared error for value/critic estimation, modified with SMDP for unprocessed datasets. Cross-entropy (LavaGap) or negative log-likelihood (UVA/Padova) for policy training.
5b	Justification for the choice of model types implemented.	15	IQL/CQL selected as state-of-the-art model-free offline RL; BC used as a supervised learning baseline.
5c	Method for evaluating the models reported in the paper, including details of train-test splits or cross-validation folds.	16	50 independent runs per experiment. UVA/Padova uses a 6-patient test cohort (30 episodes/patient); LavaGap uses 100 episodes.
5d	Method for selecting the models reported in the paper.	16	UVA/Padova: early stopping based on validation cohort performance to avoid overfitting. LavaGap: trained for fixed 10,000 update steps.
5e	For the models reported in the paper, specify details about the hyperparameter tuning:		
	Range of hyper-parameters used and a justification for why this range is reasonable.	17	Parameters were explored via a preliminary search to identify regions of stable optima across all three temporal resampling regimes.
	Method to select the best hyper-parameter configuration.	17	The final configuration was selected by confirming consistent stable performance across all dataset variants, ensuring comparisons were not biased by dataset-specific tuning.
	Specification of all hyper-parameters used to generate results reported in the paper.	18	Described in Table 1
5f	Justification that model comparisons are against appropriate baselines.	17	Compared against the PPO expert baseline (which generated the original training datasets).
**Module 6: Data leakage**			
6a	Justification that pre-processing (Section 4) and modeling (Section 5) steps only use information from the training dataset (and not the test dataset).	14	All training data and normalization parameters were calculated strictly using the training patient cohort (18-patient cohort for UVA/Padova).

Item	Description	Page	Notes
6b	Methods to address dependencies or duplicates between the training and test datasets (e.g. different samples from the same patients are kept in the same dataset partition).	12	Data leakage was prevented by partitioning the 30 virtual patients in UVA/Padova into mutually exclusive training (18), validation (6), and test (6) cohorts.
6c	Justification that each feature or input used in the model is legitimate for the task at hand and does not lead to leakage.	14	For UVA/Padova, all observations (blood glucose, carbohydrate, insulin, time of day) are from the past/present only, with no leakage of future information. Any aggregation of observations was done using the preceding window only (to avoid look-ahead bias). LavaGap uses the immediately observable pixels only.
**Module 7: Metrics and uncertainty**			
7a	All metrics used to assess and compare model performance (e.g., accuracy, AUROC etc.). Justify that the metric used to select the final model is suitable for the task.	16	Primary metric: cumulative undiscounted return (interquartile mean), representing the true live performance of the model in the environment. FQE used for off-policy estimation of the same metric.
7b	Uncertainty estimates (e.g., confidence intervals, standard deviations), and details of how these are calculated.	16	95% confidence intervals calculated using the procedure described in the Methods.
7c	Justification for the choice of statistical tests (if used) and a check for the assumptions of the statistical test.	16	No formal p-value-based hypothesis tests were used; comparison is based on non-overlapping 95% CIs as per Agarwal et al. (2021).
**Module 8: Generalizability and limitations**			
8a	Evidence of external validity.		Not reported. External validation was not performed as the study was conducted within simulated environments (UVA/Padova and LavaGap).
8b	Contexts in which the authors do not expect the study's findings to hold.	10	Results may vary in discrete clinical action spaces or high-dimensional real-world data where physiological features may be more robust to binning.
