# Supplementary material for: The impact of temporal resampling on the safety of clinical reinforcement learning: A simulated trial
Source: PLOS Digit Health. 2026 Nov 6;5(11):e0001801. doi: 10.1371/journal.pdig.0001801 (PMC13743037; doi:10.1371/journal.pdig.0001801)
Supplement: Supplementary file 3 [file pdig.0001801.s010.pdf]

Response to Reviewers

Summary of Response

Thank you for the detailed reviewer comments. We are very pleased to see that both Reviewers 1 and 2 agreed that this manuscript meets PLOS Digital Health's publication criteria. Nonetheless, their feedback has been tremendously valuable in guiding our extensive edits to improve the overall quality of the manuscript.

We have performed additional experiments on the effect of temporal binning on a real-world EHR dataset (MIMIC-IV), revealing that binning produces a large number of counterfactual artefacts (Fig 1 & 2 of our edited manuscript). We believe these artefacts, which are replicated in our simulated experiments, may contribute to the performance degradation we see following temporal resampling. We now additionally report clinically interpretable metrics of performance in our experiments (Fig S4 & S5), and have edited the Introduction and Discussion to further contextualise our paper within the field and transparently acknowledge limitations. We have further addressed their individual points, and the changes we have made in response, below.

We hope with these changes you will find the manuscript meets criteria for acceptance for publication in the Journal.

Data Availability

The simulated datasets are directly available at the provided GitHub repository. The original MIMIC-IV dataset is hosted on PhysioNet under restricted credentialled access and derived datasets cannot be openly shared without violating the data-sharing agreement. Instructions for reproducing the derived MIMIC-IV dataset for credentialled users is provided within the same GitHub repository.

Reviewer 1

1. *All core findings are derived exclusively from simulated environments. While the UVA/Padova simulator is FDA-approved for preclinical testing, it cannot replicate the complexity of real-world EHR data, which includes high-dimensional feature spaces, missing data, unmeasured confounding, heterogeneous patient populations, and complex clinical event dynamics. The generalizability of the findings to real clinical datasets remains unproven, which is a critical gap for a manuscript focused on translational clinical AI. The authours should validate their core findings in at least one real-world clinical EHR dataset*

We thank the reviewer for their feedback here, as they are absolutely correct on the limitations of simulated environments compared to complex real-world EHR data. To address this, we have included an additional set of experiments examining the effects of temporal binning on real-world EHR data derived from patients in the MIMIC-IV intensive care dataset receiving insulin infusions. We show that binning can produce a significant number of counterfactuals in this dataset (shown in Figure 2A-C), including masking hypo/hyperglycaemia, collapsing multiple decisions into a single action, and producing causally inverted event sequences. These are shown to occur with a high prevalence across a range of bin widths.

Unfortunately, the remainder of our experiments require knowledge of the true prospective performance of each ORL model (in order to compare the performance of models, and assess the accuracy of off-policy evaluation). To do so with a real-world clinical dataset would necessitate the conducting of a clinical trial. Our hypothesis, which is supported by our simulated environments, is that the artefacts introduced by temporal resampling leads to poor performance and harm. Testing this hypothesis on real patients may therefore be ethically difficult to justify in practice. Nonetheless, we feel the inclusion

of retrospective experiments to demonstrate the effects of binning on real EHR data has strengthened our overall findings, and we thank the reviewer again for raising this point.

2. *The study only evaluates model-free ORL algorithms, while model-based ORL methods are widely used in clinical research and may have pdifferential sensitivity to temporal resampling. Model-based methods learn a transition model of the clinical environment, which may be more or less robust to temporal discretization than model-free approaches. The current scope limits the generalizability of the manuscript's recommendations to only a subset of clinical ORL research. It would be better to add experimental evaluation of at least two widely used model-based ORL algorithms across all resampling conditions.*

We thank the reviewer for raising this – it is an important methodological question and one we took seriously in scoping this work. To address it directly, we carefully reviewed and categorised all 50 papers included in our literature survey by ORL approach. Of these, 45 used model-free or a combination of model-free and model-based methods. Only 4 studies used model-based methods appropriate to an offline setting, and critically, all 4 employed bespoke approaches rather than any standardised or widely-adopted technique. A further 5 papers incorporated model-based elements but used methods that are not suitable for a purely offline setting (e.g., policy iteration) and therefore fall outside the scope of this work.

This distribution reflects the current state of clinical ORL research: model-based offline methods remain a small and methodologically heterogeneous minority. Unlike model-free algorithms – where clear community benchmarks like IQL and CQL exist – there is no established model-based equivalent against which a fair and reproducible comparison could be constructed. However, we recognise this represents a genuine limitation and have made this clear in our discussion section. We have also updated Supplementary Table S1 to systematically label the ORL approaches across all 50 reviewed papers, and have updated our introduction to also clarify this rationale.

Reviewer 2

The paper's topic is important, focusing on the potential safety risks of temporal resampling in clinical offline reinforcement learning. The authors conducted a prospective simulation evaluation using the UVA/Padova diabetes simulator, showing that binning and interpolation degrade model performance, and that retrospective FQE evaluations may overestimate true performance. Overall, the research question is valuable, and the experimental framework has potential; however, the current conclusions exceed existing evidence, and further key controls and more clinically meaningful outcome measures are needed.

1. *The core causal conclusions are still insufficient. The paper argues that the performance degradation is mainly caused by temporal resampling, but the current comparison simultaneously alters multiple factors. Untreated data used the SMDP framework, while binned and interpolated data used the standard MDP framework. Therefore, the results reflect not only whether the data was resampled but also differences in the modeling framework, action duration, reward accumulation method, and sample structure. It is recommended that the authors supplement key control experiments, such as using MDP for untreated data, adding time interval information to binned data and using SMDP, or simply changing whether binning is used within a fixed learning framework. Otherwise, the conclusion should converge to: under the settings of this paper, common resampling processes are associated with performance degradation, rather than directly proving that resampling itself necessarily causes safety risks.*

We appreciate the reviewer's careful and thoughtful consideration of our work, and the nuance behind their points, which has prompted several necessary changes to our written work. First and foremost, we realise that our original submission was ambiguous regarding the use of MDP and SMDP frameworks. We would like to clarify that, strictly speaking, **all** of our experiments use an SMDP approach – but

it is also the case that SMDPs are functionally and mathematically equivalent to MDPs when fixed intervals are used. We have made this distinction clearer when discussing our results.

We agree with several of the reviewer's points. Our core claim is as follows: the practice of temporally resampling involves the simultaneous alteration of multiple factors. This includes a simplified observation state space, action dynamics, and altered time interval information provided to the model. This combination of processes leads to degraded model performance and retrospective evaluation accuracy, as demonstrated by our results. We have rewritten sections of our introduction and discussion to better clarify this, and added experiments that further demonstrate how binning corrupts the training dataset. We also hope that our use of different binning widths provides some degree of control, as suggested in the reviewer's comments.

The reviewer has also suggested "adding time interval information to binned data and using SMDP". However, we were not able to identify a clear way to achieve this, as each fixed time bin can contain multiple actions – as a result, we do not believe there is a standard way to define the interval between bins (besides the fixed window size used in our experiments).

2. *The simulation of irregular clinical decision-making is still relatively simplified. The paper emphasizes that clinical decision-making is event-driven, but in the UVA/Padova irregular environment, the decision interval is randomly sampled from 1 to 12 time steps. This setting does not reflect the impact of changes in patient condition, glycemic risk, eating events, or treatment responses on decision frequency, and therefore still differs significantly from the rhythm of real clinical decision-making. It is recommended that the authors add a state-related interval mechanism, such as shortening the decision interval when blood glucose changes rapidly or approaches a dangerous range, and lengthening the interval when it is stable. If this cannot be added, it should be clearly stated in the abstract and discussion that this paper studies random irregular intervals, rather than fully simulating real clinical workflows.*

We thank the reviewer for bringing this to our attention. It is correct that our sampling distribution is from a state-independent uniform distribution rather than a state-dependent one, which we felt added too much confounding in its specific parameterisation to appropriately justify. We have stated this more transparently in the discussion.

3. *Safety conclusions need more clinically interpretable indicators to support them. Currently, the main results rely on standardized average rewards. This indicator is suitable for comparison of reinforcement learning algorithms, but is insufficient to support conclusions regarding patient safety. For diabetes control tasks, authors are advised to supplement with more intuitive clinical indicators, such as the proportion of time blood glucose within the target range, the proportion of time with hypoglycemia and hyperglycemia, severe hypoglycemic and hyperglycemic events, the proportion of early discontinuation, insulin dose distribution, and performance in adult, adolescent, and pediatric subgroups. If the paper argues that time resampling poses a patient safety risk, it should demonstrate the specific manifestations of these risks in clinical indicators, rather than relying primarily on reinforcement learning reward values.*

The reviewer makes an important point which we have attempted to address in our revised manuscript. In our experiments, the reward function is calculated from clinically relevant outcomes (the combination of the Magni risk index, plus severe penalties for extreme hypo/hyperglycaemia). To examine how changes in reward correlate with clinical markers, we have included additional plots illustrating each model's 1) proportion of time spent in target glucose range (pTIR) and 2) proportion of episodes that terminate early (pET) due to extreme hypo- or hyperglycaemia. We found that **only** CQL models trained on unprocessed data were able to improve **both** their pTIR and pET. IQL models trained on unprocessed data improved their pET and maintained the baseline pTIR. All other datasets led to a

worsening of one or both metrics, demonstrating the increased patient safety risk through the use of clinical metrics.

4. *Retrospective assessment results are important, but require more comprehensive methodological interpretation. The overestimation of binning model performance by FQE is one of the paper's most valuable findings, but its origin needs further explanation. FQE inaccuracies may stem from spurious trajectories caused by binning, from the target strategy exceeding the data support range, or from the model's own specifications. Authors are advised to supplement with the following: explain why the FQE discount factor differs from the training discount factor; report the action differences or coverage between the target strategy and the behavioral strategy; add at least one additional offline assessment method for sensitivity analysis; and explain whether FQE maintains the same inaccuracy pattern on independent patient data. This will more strongly support the conclusion that retrospective assessment may mask safety risks.*

We thank the reviewer again for their suggestions, which we have incorporated into the revised manuscript. We have rewritten the discussion to better highlight our claim that FQE inaccuracies stem from spurious trajectories caused by binning – which in turn result in the target strategy learning harmful out-of-distribution behaviours (only revealed during prospective model evaluation). This effect is not seen in ORL models trained on interpolated data, which lack the counterfactual artefacts described in binned data and therefore have well-calibrated FQE predictions.

We gave careful consideration to the inclusion of additional offline evaluation methods. The commonly used alternatives to FQE for off-policy evaluation involve importance sampling (IS) (e.g., WIS, doubly robust). However, our evaluation target is a deterministic policy over a continuous action space, for which per-decision importance ratios are undefined. Whilst we could change our policy to a stochastic one to include IS methods, in practice it seems very unlikely that any real-world healthcare setting/ clinical trial would allow a deployed policy to be stochastic rather than deterministic (for safety/ethical reasons).

The reviewer has suggested evaluating FQE on "independent patient data" – we are pleased to confirm that in our original set of experiments, we used an isolated test cohort of independent patients for both the retrospective and prospective evaluations. This cohort is entirely unseen and separate from the cohort of patients used to train the FQE and ORL models. We have also updated our methods section to explain that the FQE discount factor is set to 1.0 so that the output directly reflects the undiscounted cumulative episodic return, allowing a proper comparison with prospective episodic returns.

We would like to thank both reviewers again for their careful consideration of our work and their valued feedback, which has led to several changes that we feel significantly improved the quality of our resubmitted manuscript.
