# Supplementary material for: The impact of temporal resampling on the safety of clinical reinforcement learning: A simulated trial
Source: PLOS Digit Health. 2026 Nov 6;5(11):e0001801. doi: 10.1371/journal.pdig.0001801 (PMC13743037; doi:10.1371/journal.pdig.0001801)
Supplement: Peer review history

# Peer review: Decision Letter 0

**DOI:** 10.1371/journal.pdig.0001801.r001

**Authors:** Zhenwei Shi, Wei Zhang

26 May 2026

Response to Reviewers Revised Manuscript with Track Changes Manuscript **Journal Requirements:**

1. We ask that a manuscript source file is provided at Revision. Please upload your manuscript file as a .doc, .docx, .rtf or .tex.

2. Please upload separate figure files in .tif or .eps format. Also, remove the figures from your manuscript file but keep the legends.

For more information about figure files please see our guidelines:

https://journals.plos.org/digitalhealth/s/figures

https://journals.plos.org/digitalhealth/s/figures#loc-file-requirements

**Additional Editor Comments (if provided):** **Reviewers' Comments:**

**Comments to the Author**

1. Does this manuscript meet PLOS Digital Health’s publication criteria?

Reviewer #1: Yes

Reviewer #2: Yes

**********

2. Has the statistical analysis been performed appropriately and rigorously?

Reviewer #1: Yes

Reviewer #2: Yes

**********

3. Have the authors made all data underlying the findings in their manuscript fully available (please refer to the Data Availability Statement at the start of the manuscript PDF file)?

The PLOS Data policy

Reviewer #1: Yes

Reviewer #2: Yes

**********

4. Is the manuscript presented in an intelligible fashion and written in standard English?

Reviewer #1: Yes

Reviewer #2: Yes

**********

**Reviewer #1:**  1.All core findings are derived exclusively from simulated environments. While the UVA/Padova simulator is FDA-approved for preclinical testing, it cannot replicate the complexity of real-world EHR data, which includes high-dimensional feature spaces, missing data, unmeasured confounding, heterogeneous patient populations, and complex clinical event dynamics. The generalizability of the findings to real clinical datasets remains unproven, which is a critical gap for a manuscript focused on translational clinical AI. The authours should validate their core findings in at least one real-world clinical EHR dataset

2.The study only evaluates model-free ORL algorithms, while model-based ORL methods are widely used in clinical research and may have differential sensitivity to temporal resampling. Model-based methods learn a transition model of the clinical environment, which may be more or less robust to temporal discretization than model-free approaches. The current scope limits the generalizability of the manuscript’s recommendations to only a subset of clinical ORL research. It would be better to add experimental evaluation of at least two widely used model-based ORL algorithms across all resampling conditions.

**Reviewer #2:**  The paper's topic is important, focusing on the potential safety risks of temporal resampling in clinical offline reinforcement learning. The authors conducted a prospective simulation evaluation using the UVA/Padova diabetes simulator, showing that binning and interpolation degrade model performance, and that retrospective FQE evaluations may overestimate true performance. Overall, the research question is valuable, and the experimental framework has potential; however, the current conclusions exceed existing evidence, and further key controls and more clinically meaningful outcome measures are needed.

1. The core causal conclusions are still insufficient. The paper argues that the performance degradation is mainly caused by temporal resampling, but the current comparison simultaneously alters multiple factors. Untreated data used the SMDP framework, while binned and interpolated data used the standard MDP framework. Therefore, the results reflect not only whether the data was resampled but also differences in the modeling framework, action duration, reward accumulation method, and sample structure. It is recommended that the authors supplement key control experiments, such as using MDP for untreated data, adding time interval information to binned data and using SMDP, or simply changing whether binning is used within a fixed learning framework. Otherwise, the conclusion should converge to: under the settings of this paper, common resampling processes are associated with performance degradation, rather than directly proving that resampling itself necessarily causes safety risks.

2. The simulation of irregular clinical decision-making is still relatively simplified. The paper emphasizes that clinical decision-making is event-driven, but in the UVA/Padova irregular environment, the decision interval is randomly sampled from 1 to 12 time steps. This setting does not reflect the impact of changes in patient condition, glycemic risk, eating events, or treatment responses on decision frequency, and therefore still differs significantly from the rhythm of real clinical decision-making. It is recommended that the authors add a state-related interval mechanism, such as shortening the decision interval when blood glucose changes rapidly or approaches a dangerous range, and lengthening the interval when it is stable. If this cannot be added, it should be clearly stated in the abstract and discussion that this paper studies random irregular intervals, rather than fully simulating real clinical workflows.

3. Safety conclusions need more clinically interpretable indicators to support them. Currently, the main results rely on standardized average rewards. This indicator is suitable for comparison of reinforcement learning algorithms, but is insufficient to support conclusions regarding patient safety. For diabetes control tasks, authors are advised to supplement with more intuitive clinical indicators, such as the proportion of time blood glucose within the target range, the proportion of time with hypoglycemia and hyperglycemia, severe hypoglycemic and hyperglycemic events, the proportion of early discontinuation, insulin dose distribution, and performance in adult, adolescent, and pediatric subgroups. If the paper argues that time resampling poses a patient safety risk, it should demonstrate the specific manifestations of these risks in clinical indicators, rather than relying primarily on reinforcement learning reward values.

4. Retrospective assessment results are important, but require more comprehensive methodological interpretation. The overestimation of binning model performance by FQE is one of the paper's most valuable findings, but its origin needs further explanation. FQE inaccuracies may stem from spurious trajectories caused by binning, from the target strategy exceeding the data support range, or from the model's own specifications. Authors are advised to supplement with the following: explain why the FQE discount factor differs from the training discount factor; report the action differences or coverage between the target strategy and the behavioral strategy; add at least one additional offline assessment method for sensitivity analysis; and explain whether FQE maintains the same inaccuracy pattern on independent patient data. This will more strongly support the conclusion that retrospective assessment may mask safety risks.

**********

what does this mean?). If published, this will include your full peer review and any attached files.

**Do you want your identity to be public for this peer review?**  If you choose “no”, your identity will remain anonymous but your review may still be made public.

For information about this choice, including consent withdrawal, please see our Privacy Policy

Reviewer #1: No

Reviewer #2: No

**********

**Figure resubmission:**

**Reproducibility:**

To enhance the reproducibility of your results, we recommend that authors of applicable studies deposit laboratory protocols in protocols.io, where a protocol can be assigned its own identifier (DOI) such that it can be cited independently in the future. Additionally, PLOS ONE offers an option to publish peer-reviewed clinical study protocols. Read more information on sharing protocols at https://plos.org/protocols?utm_medium=editorial-email&utm_source=authorletters&utm_campaign=protocols

# Author comment: Author response to Decision Letter 1

**DOI:** 10.1371/journal.pdig.0001801.r002

23 Jul 2026

Attachment Submitted filename: response_to_reviewers.pdf

# Peer review: Decision Letter 1

**DOI:** 10.1371/journal.pdig.0001801.r003

**Authors:** Zhenwei Shi

2 Oct 2026

The impact of temporal resampling on the safety of clinical reinforcement learning: A simulated trial

PDIG-D-26-00382R1

Dear Dr Frost,

We are pleased to inform you that your manuscript 'The impact of temporal resampling on the safety of clinical reinforcement learning: A simulated trial' has been provisionally accepted for publication in PLOS Digital Health.

Before your manuscript can be formally accepted you will need to complete some formatting changes, which you will receive in a follow-up email from a member of our team.

Please note that your manuscript will not be scheduled for publication until you have made the required changes, so a swift response is appreciated.

IMPORTANT: The editorial review process is now complete. PLOS will only permit corrections to spelling, formatting or significant scientific errors from this point onwards. Requests for major changes, or any which affect the scientific understanding of your work, will cause delays to the publication date of your manuscript.

If your institution or institutions have a press office, please notify them about your upcoming paper to help maximize its impact. If they'll be preparing press materials, please inform our press team as soon as possible -- no later than 48 hours after receiving the formal acceptance. Your manuscript will remain under strict press embargo until 2 pm Eastern Time on the date of publication. For more information, please contact digitalhealth@plos.org.

Thank you again for supporting Open Access publishing; we are looking forward to publishing your work in PLOS Digital Health.

Best regards,

Zhenwei Shi, PhD

Section Editor

PLOS Digital Health

***********************************************************

**Additional Editor Comments (if provided):**

**Reviewer Comments (if any, and for reference):**

Reviewer's Responses to Questions

**Comments to the Author**

Reviewer #1: All comments have been addressed

**********

publication criteria?

Reviewer #1: Yes

**********

3. Has the statistical analysis been performed appropriately and rigorously?

Reviewer #1: Yes

**********

4. Have the authors made all data underlying the findings in their manuscript fully available (please refer to the Data Availability Statement at the start of the manuscript PDF file)?

The PLOS Data policy

Reviewer #1: Yes

**********

5. Is the manuscript presented in an intelligible fashion and written in standard English?

Reviewer #1: Yes

**********

Reviewer #1: All my comments have been well addressed, and I have no more questions.

**********

what does this mean?). If published, this will include your full peer review and any attached files.

**Do you want your identity to be public for this peer review?**  If you choose “no”, your identity will remain anonymous but your review may still be made public.

For information about this choice, including consent withdrawal, please see our Privacy Policy

Reviewer #1: No

**********
